# Triphosphate Tunnel Metalloenzyme 2 Acts as a Downstream Factor of ABI4 in ABA-Mediated Seed Germination

**DOI:** 10.3390/ijms24108994

**Published:** 2023-05-19

**Authors:** Yu-Rui Feng, Ting-Ting Li, Shi-Jia Wang, Ying-Tang Lu, Ting-Ting Yuan

**Affiliations:** 1 State Key Laboratory of Hybrid Rice, College of Life Sciences, Wuhan University, Wuhan 430072, China; 2 Jiangsu Key Laboratory of Marine Pharmaceutical Compound Screening, Jiangsu Ocean University, Lianyungang 222005, China

**Keywords:** TTM2, seed germination, ABA, ABI4

## Abstract

Seed germination is a complex process that is regulated by various exogenous and endogenous factors, in which abscisic acid (ABA) plays a crucial role. The triphosphate tunnel metalloenzyme (TTM) superfamily exists in all living organisms, but research on its biological role is limited. Here, we reveal that *TTM2* functions in ABA-mediated seed germination. Our study indicates that *TTM2* expression is enhanced but repressed by ABA during seed germination. Promoted *TTM2* expression in *35S::TTM2-FLAG* rescues ABA-mediated inhibition of seed germination and early seedling development and *ttm2* mutants exhibit lower seed germination rate and reduced cotyledon greening compared with the wild type, revealing that the repression of *TTM2* expression is required for ABA-mediated inhibition of seed germination and early seedling development. Further, ABA inhibits *TTM2* expression by ABA insensitive 4 (ABI4) binding of *TTM2* promoter and the ABA-insensitive phenotype of *abi4-1* with higher *TTM2* expression can be rescued by mutation of *TTM2* in *abi4-1 ttm2-1* mutant, indicating that *TTM2* acts downstream of *ABI4*. In addition, *TTM1*, a homolog of *TTM2*, is not involved in ABA-mediated regulation of seed germination. In summary, our findings reveal that TTM2 acts as a downstream factor of ABI4 in ABA-mediated seed germination and early seedling growth.

## 1. Introduction

Seed germination, an early event in plant development, has a significant impact on the later vegetative and reproductive growth of plants [1]. Seeds are induced into primary dormancy after maturation and require prolonged dry storage or low-temperature water absorption (stratification) to release dormancy for germination under favorable environmental conditions [2,3]. Seed germination is regulated by various exogenous and endogenous factors [4,5,6] Among these factors, abscisic acid (ABA) and gibberellin (GA) are the major players associated with seed dormancy and germination, respectively [7,8], and the balance between these two hormones dictates the seeds germination ability [3].

Previous studies displayed that ABA content decreased rapidly after cold stratification of seeds [9] and exogenous application of ABA inhibits seed germination [10,11]. Mutation in *CYP707As,* key catabolism genes of ABA, results in higher ABA accumulation and leads to delayed seed germination compared with the wild type [12]. Further, ABA modulates the expression of numerous germination genes through its signaling pathways, such as *EM1* and *EM6* [13,14]. ABA is identified by its receptor PYR/PYL/RCAR family, and the ABA-receptor complex can bind to PP2Cs, thus inhibiting their activity and leading to the activation of SnRK2s and turning on ABA signaling [15,16,17]. ABA insensitive 3 (ABI3), ABI4, and ABI5 are downstream factors of ABA signaling, and all mutants of *abi3*, *abi4*, and *abi5* exhibit insensitivity to ABA in seed germination [18,19,20].

ABI4 participates in numerous facets of plant growth and development, but it has been most widely studied in seed germination and development [21,22,23,24]. It has been documented that many factors regulate *ABI4* expression [25,26]. For example, both WRKY6 and MYB96 promote, but NDX, WRKY18, WRKY40, WRKY60, BASS2, and OS3 repress *ABI4* expression during seed germination [27,28,29,30,31,32,33]. As a multifunctional transcription factor, there are also many genes whose expression is regulated by ABI4 such as *CYP707As* and *CHO1* in seed germination [18,34,35].

TTM superfamily exists in all domains of life and hydrolyzes a range of organophosphate substrates, which comprise a group of enzymes [36]. This family has three members in *Arabidopsis*: TTM1, TTM2, and TTM3. TTM1 and TTM2 contain both the CYTH domain and P-cycle kinase/uric acid kinase domain, but TTM3 consists only CYTH domain [37,38,39]. Although there is a high sequence similarity between TTM1 and TTM2, these two members function differently in plants. For example, TTM1 but not TTM2 plays an active role in regulating plant leaf senescence. When treated with ABA or darkness, TTM1 phosphorylation was increased and the senescence-associated programmed cell death was accelerated [37,40]. TTM2 plays a negative role in plant resistance to oomycete response as *ttm2* mutants exhibit enhanced hypersensitive response and increased salicylic acid accumulation, but TTM1 is not involved in plant immunity and has no synergistic effect with TTM2 [37]. Whether and how TTM2 functions in plant growth and development remain unclear.

Here, the function of TTM2 in ABA-dependent seed germination is reported. Our study shows that ABA inhibits *TTM2* expression during seed germination. The *ttm2* mutants show hypersensitivity to ABA-mediated seed germination, while *35S::TTM2-FLAG* plants exhibited less insensitivity to ABA-mediated seed germination. ABA core transcription factor ABI4 represses the transcription level of *TTM2* by directly binding to its promoter, and ABA-insensitive germination of *abi4-1* is restored by crossing *abi4-1* with *ttm2-1* ablating *TTM2*, demonstrating that *TTM2* is a downstream target of *ABI4*. In addition, there is a non-redundant function between *TTM2* and *TTM1* in regulating seed germination. In conclusion, our results indicate that *TTM2* is regulated by ABI4 and contributes to ABA-mediated seed germination and early seedling growth.

## 2. Results

### 2.1. ABA Regulates TTM2 Expression during Seed Germination

**Figure 1:**
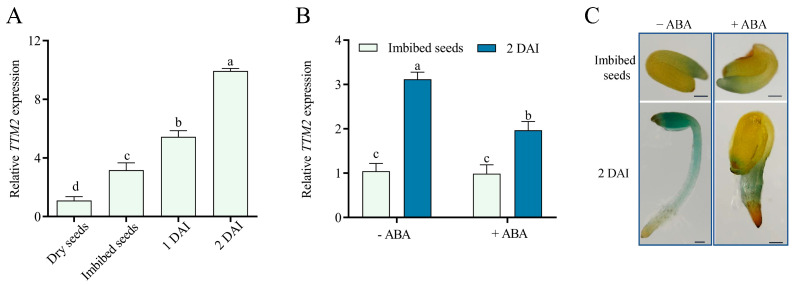
*TTM2* expression changes during seed germination in response to ABA. (**A**) Expression of *TTM2* in wild-type dry seeds, imbibed seeds, and germinating seeds (1 DAI and 2 DAI). Analysis of *TTM2* transcript levels by qRT-PCR. The wild-type (WT) seeds were soaked at 4 °C for 3 d in water and grown on 1/2 Murashige & Skoog (MS) medium at various times, then harvested the plants at the specified dates. DAI, days after imbibition. The *ACT2* gene was applied as an internal control. Expression levels were normalized to dry seeds, which was set at 1. Data are shown as mean ± SD (*n* = 3). Various letters represent significant differences at *p* < 0.05 by one-way ANOVA and Tukey’s multiple comparison test. (**B**) The qRT-PCR to determine the expression of *TTM2*. Imbibed seeds of the wild type were planted in 1/2 MS medium (-ABA) or 1/2 MS medium containing 0.5 μM ABA (+ABA) for 2 days followed by RNA extraction. The *ACT2* gene was applied as an internal control. Expression levels were normalized to those of untreated (-ABA) imbibed seeds, which was set at 1. Data are shown as mean ± SD (*n* = 3). Various letters represent significant differences at *p* < 0.05 by one-way ANOVA with Tukey’s multiple comparison test. (**C**) GUS staining assay for *ProTTM2::GUS* #1 lines. Imbibed seeds were planted on 1/2 MS medium (-ABA) or 1/2 MS medium containing 0.5 μM ABA (+ABA) for 2 days, and then germinating seeds were subjected to GUS staining. Bars = 100 μm.

While whether and how TTM2 functions in plant growth and development remain unclear, the reported public microarray data showed that *TTM2* expression is elevated during seed imbibition [41]. Thus, we used the quantitative real-time PCR (qRT-PCR) to assay transcription levels of *TTM2* in dry seeds, imbibed seeds, and germinating seeds on 1/2 Murashige and Skoog (MS) medium (1 day after imbibition, DAI, and 2 DAI). Our data demonstrated that *TTM2* expression was markedly increased in imbibed seeds and germinating seeds compared to dry seeds (Figure 1A). Due to the high homology of *TTM2* with *TTM1* and *TTM3*, we investigated their transcription levels during germination and early seedling development. The results showed that *TTM1* and *TTM3* were not significantly altered during seed germination, which further confirmed the functional diversification of this family (Figure S1).

Considering that ABA, one of the main hormones regulating seed germination, can inhibit seed germination [3,7,9], we also investigated whether ABA is associated with *TTM2* expression in seed germination. We observed that promoted *TTM2* expression in the seeds on 1/2 MS medium was repressed on 1/2 MS medium containing 0.5 μM ABA (Figure 1B and Figure S2). In addition, we obtained *ProTTM2::GUS* transgenic plants, in which GUS is under the control of the *TTM2* promoter (Figure S3). Consistently, GUS staining assays showed that GUS expression in germinating seeds on 1/2 MS medium containing 0.5 μM ABA was much lower than that in untreated germinating seeds (Figure 1C). The findings imply that *TTM2* may be associated with ABA-inhibited seed germination.

Since seed germination is severely regulated with mutual antagonism between ABA and GA [42,43,44]. Therefore, we also tested whether *TTM2* expression was regulated by GA during seed germination, and found that, unlike ABA, the transcriptional level of *TTM2* was not associated with exogenous GA treatment (Figure S4).

### 2.2. Repression of TTM2 Expression Is Required for ABA-Mediated Inhibition of Seed Germination

**Figure 2:**
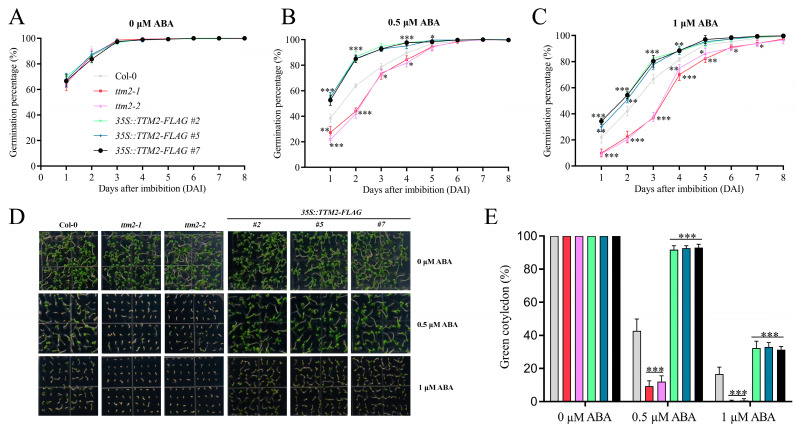
ABA-mediated inhibition of seed germination is aggravated in *ttm2* mutants but attenuated in *TTM2*-overexpressing lines. (**A**–**C**) Seed germination curves. The imbibed *Arabidopsis* WT (Col-0), knockout mutants (*ttm2-1*, *ttm2-2*), and *TTM2* overexpression plants (#2, #5, #7) transferred to 1/2 MS (**A**) or 1/2 MS medium containing 0.5 μM ABA (**B**) or 1 μM ABA (**C**) for germination experiments and then calculated at the indicated times. (**D**) Seed germination phenotypes. Imbibed seeds were germinated and grown on 1/2 MS medium with 0, 0.5, or 1 μM ABA for 8 days. (**E**) Cotyledon greening rate. The green cotyledon rate was measured as (**D**). Each replicate had at least 100 seeds counted in it. Data are shown as mean ± SD (*n* = 3). Asterisk denotes a statistically significant difference compared with their wild type at indicated times. *, *p* < 0.05; **, *p* < 0.01; ***, *p* < 0.001 (Student’s *t*-test).

Then, we investigated whether changes in *TTM2* expression can affect ABA-mediated seed germination. Therefore, we generated *35S::TTM2-FLAG* transgenic lines (Figure S3) and assessed their seed germination when treated with ABA. Under normal conditions, the *35S::TTM2-FLAG* transgenic plants had similar germination rates to the wild type. However, when germinated on 1/2 MS medium containing 0.5 or 1 μM ABA, compared with the wild type, the germination rate of *35S::TTM2-FLAG* was higher (Figure 2A–C). Consistent with the germination rate, in the absence of ABA, cotyledon greening of *35S::TTM2-FLAG* plants had no difference from the wild type. In the presence of ABA, *35S::TTM2-FLAG* plants were hyposensitive to ABA by displaying increased cotyledon-greening percentages compared with the wild type (Figure 2D,E). Combined with the data above that ABA suppresses the expression of *TTM2*, these results reveal that the repression of *TTM2* expression is needed for ABA-mediated inhibition of seed germination and early seedling development.

This conclusion was further supported by assaying seed germination rate and cotyledon greening of *ttm2-1* (SALK_145897) and *ttm2-2* (SALK_114669), two previously reported T-DNA insertion knockout mutants [38]. When germinated and grown on 1/2 MS medium without ABA, the germination rates and cotyledon greening of these two mutants had no obvious difference from the wild type. However, when 1/2 MS medium containing 0.5 or 1 μM ABA, both *ttm2-1* and *ttm2-2* exhibited lower seed germination rate and reduced cotyledon greening in comparison to the wild type. And the *ttm2* mutants, which were hypersensitive to ABA, had almost no green cotyledons at 8 DAI when treated with 1 μM ABA (Figure 2). In our further experiments, 0.5 μM ABA was used for treatment.

### 2.3. TTM2 Acts Downstream of ABI4 to Regulate Seed Germination

**Figure 3:**
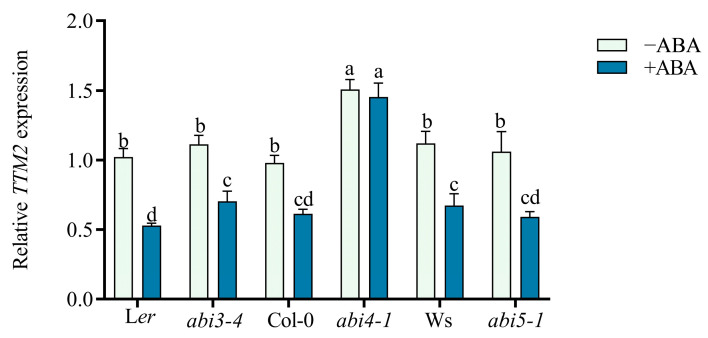
The repression of *TTM2* expression in response to ABA is abolished in *abi4-1*. Imbibed seeds of different genotypes were germinated on 1/2 MS medium (−ABA) or 1/2 MS medium with 0.5 μM ABA (+ABA) for 2 days. Harvested plants were subjected to RNA extraction and qRT-PCR analysis. Among them, Landsberg *erecta* (L*er*) is the wild-type control for *abi3-4*; Columbia (Col-0) is the wild-type control for *abi4-1*; Wassilewskija (Ws) is the wild-type control for *abi5-1*. The *ACT2* gene was applied as an internal control. Expression levels were normalized to those of untreated (−ABA) Col-0 plants, which was set at 1. Data are shown as mean ± SD (*n* = 3). Various letters represent significant differences at *p* < 0.05 by one-way ANOVA with Tukey’s multiple comparison test.

*ABI* genes are the critical components of the ABA signaling response. Among them, ABI3, ABI4, and ABI5 have been identified as core transcription factors in seed germination [4,18,45]. To investigate how ABA regulates *TTM2*, we examined *TTM2* transcriptional levels in *abi* mutants. We found that *TTM2* expression can be still repressed by ABA in either *abi3-4* or *abi5-1* compared to the wild type, while the inhibition of *TTM2* transcription by ABA in the wild type was abolished in *abi4-1* (Figure 3 and Figure S5), indicating ABI4 is essential for ABA-mediated inhibition of *TTM2* expression.

**Figure 4:**
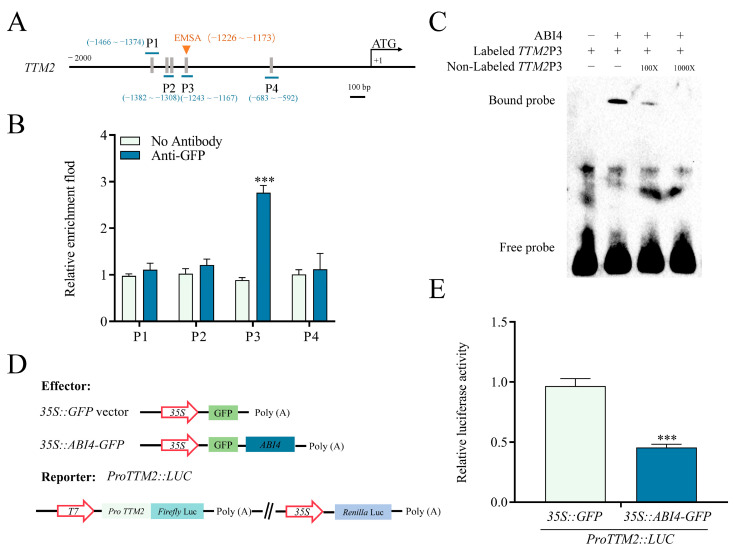
ABI4 regulates *TTM2* expression by directly binding to its promoter. (**A**) Promoters of *TTM2* were analyzed. The *TTM2* promoter region is 2000 bp upstream of ATG. ATG, is the start codon. The CCAC motifs are marked with gray boxes. The blue line represents the fragments amplified in the ChIP assay. The orange triangle represents the location of the probe used for EMSA. (**B**) ChIP-qPCR enrichment of the specified DNA fragments using anti-GFP antibodies. Chromatin from *35S::ABI4-GFP* plants. The values of ChIP were normalized to their respective inputs. The *UBQ5* was used as a negative control. (**C**) EMSA of ABI4 bounding to *TTM2* promoter (*TTM2-P3*) *in vitro*. The purified ABI4 protein was incubated with biotin-labeled TTM2-P3 probes. The unlabeled probe was used as a competitor. (**D**) A schematic of vector constructs used in a dual-luciferase reporter assay. Effector: *35S::ABI4-GFP*. Reporter: *ProTTM2::LUC*. (**E**) Quantitative analyses of luminescence intensity. At least three biological replicates of the above experiment were performed. Data are shown as mean ± SD (*n* = 3). Asterisks indicate significant differences. ***, *p* < 0.001 (Student’s *t*-test).

To explore how ABI4 modulates *TTM2* expression, we first searched for the CCAC box, an important element in ABI4 target gene promoters [18], in the *TTM2* promoter and found five CCAC boxes (Figure 4A). Then, we generated *35S::ABI4-GFP* transgenic plants (Figure S6) and used them for chromatin immunoprecipitation (ChIP). Our findings demonstrated that the P3 region of the *TTM2* promoter was substantially enriched by anti-GFP in chromatin immunoprecipitated with *35S::ABI4-GFP*, indicating ABI4 binding of the P3 region (Figure 4B). Our DNA electrophoretic mobility shift assays (EMSAs) utilizing ABI4 protein produced and purified from *E. coli* further confirmed this binding. Our results found that the ABI4 protein bound to the P3 probe that was biotin-labeled, while this binding could be completed by an unlabeled version of the DNA probe (Figure 4C). To further substantiate the impact of ABI4 on the modulation of *TTM2* expression, we also detected the expression of *TTM2* with dual luciferase assays in *Nicotiana benthamiana* leaves. In these experiments, the reporter vector consists of a firefly luciferase (*LUC*) gene driven by the *TTM2* promoter, the *ABI4* gene driven by the *CaMV 35S* was generated for the effector vector (Figure 4D) and LUC/REN ratio in the dual-LUC assay indicated relative LUC activity. The analysis displayed that the co-expression of *35S::ABI4-GFP* and *ProTTM2::LUC* markedly reduced LUC activity in comparison with the *35S:: GFP* and *ProTTM2::LUC* co-expression (Figure 4E). Taken together, the tests indicated that ABI4 is a transcriptional repressor of *TTM2*.

**Figure 5:**
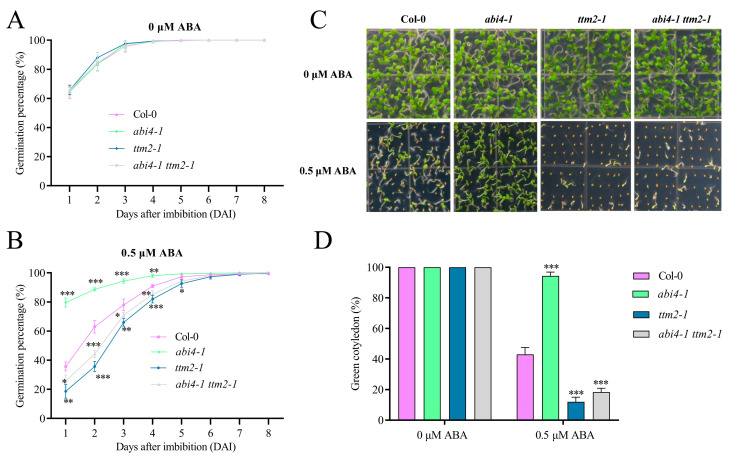
The ABA-insensitive phenotype of the *abi4-1* mutant is rescued by *ttm2-1*. (**A**, **B**) Seed germination curves. Germination rates are for imbibed *Arabidopsis* WT (Col-0), *abi4-1* mutants, *ttm2-1* mutants, and *abi4-1 ttm2-1* double mutant planted on 1/2 MS medium (**A**) or 1/2 MS medium with 0.5 μM ABA (**B**) for germination experiments and then calculated at the indicated times. (**C**) Seed germination phenotypes. Imbibed seeds were incubated on 1/2 MS medium containing 0 or 0.5 μM ABA for 8 days. (**D**) Cotyledon greening rate. The green cotyledon rate was measured as (**C**). Each replicate had at least 100 seeds counted in it. Data are shown as mean ± SD (*n* = 3). Asterisk denotes a statistically significant difference compared with their wild type at indicated times. *, *p* < 0.05; **, *p* < 0.01; ***, *p* < 0.001 (Student’s *t*-test).

To characterize the genetic relationship between *ABI4* and *TTM2*, we obtained the *abi4-1 ttm2-1* double mutant by crossing *abi4-1* with *ttm2-1*. When germinated on 1/2 MS medium, all genotypes including the wild type, *abi4-1*, *ttm2-1*, and *abi4-1 ttm2-1* showed similar germination rates (Figure 5A). When subjected to ABA treatment, the seed germination rate of the *abi4-1* mutant was higher than that of the wild type as previously reported [46], whereas the *abi4-1 ttm2-1* mutant, similar to the *ttm2-1* mutant, exhibited a lower germination rate compared with the wild type (Figure 5B). Similarly, both *ttm2-1* and *abi4-1 ttm2-1* mutants showed reduced cotyledon greening compared with the wild type when grown on 1/2 MS medium containing 0.5 μM ABA, whereas the cotyledon greening was higher in the *abi4-1* mutant than the wild type (Figure 5C,D). Combined with our data above that ABI4 modulates *TTM2* expression by directly binding to its promoter, these findings indicate that *TTM2* is located genetically downstream of *ABI4* in ABA-mediated inhibition of seed germination and early seedling development.

### 2.4. TTM1 Is Not Involved in ABA-Mediated Seed Germination and Early Seedling Development

**Figure 6:**
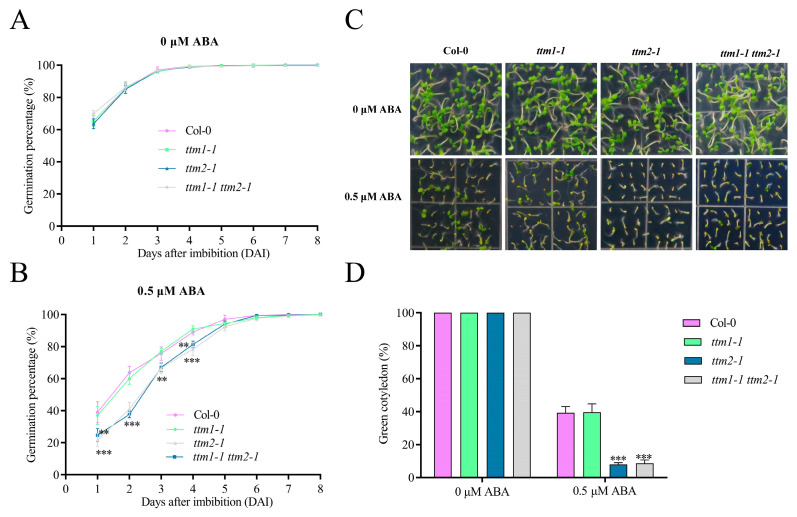
The *ttm2-1* and *ttm1-1 ttm2-1* mutants exhibited similar responses to ABA during seed germination. (**A**,**B**) Seed germination curves. Germination rates are for imbibed *Arabidopsis* WT (Col-0), knockout mutants (*ttm1-1*, *ttm2-1*), and double mutant *ttm1-1 ttm2-1* planted to 1/2 MS (**A**) or 1/2 MS medium with 0.5 μM ABA (**B**) for germination experiments and then calculated at the indicated times. (**C**) Seed germination phenotypes. Imbibed seeds were incubated on 1/2 MS medium containing 0 or 0.5 μM ABA for 8 days. (**D**) Cotyledon greening rate. The green cotyledon rate was measured as (**C**). Each replicate had at least 100 seeds counted in it. Data are shown as mean ± SD (*n* = 3). Asterisk denotes a statistically significant difference compared with their wild type at indicated times. **, *p* < 0.01; ***, *p* < 0.001 (Student’s *t*-test).

TTM1 and TTM2 belong to the TTM family with a high degree of amino acid sequence similarity, however, these two members function differently in plants [38]. Here, we employed the *ttm1-1* mutant (SALK_079237), a previously reported T-DNA insertion knockout line, to explore whether TTM1 acts in ABA-mediated seed germination and early seedling development [37]. Our outcomes indicated that the *ttm1-1* mutant showed similar germination rates and cotyledon-greening percentages to the wild type when germinated and grown on 1/2 MS medium in the presence or absence of ABA (Figure 6). Then, we tried to test whether TTM1 has a possible synergistic effect with TTM2. Thus, we obtained the *ttm1-1 ttm2-1* mutant (Figure S7) and examined germination genotypes. All tested genotypes including the wild type, *ttm1-1*, *ttm2-1*, and *ttm1-1 ttm2* showed similar germination and cotyledon-greening rates when germinated and grown on 1/2 MS medium without ABA (Figure 6A). When ABA was applied, the *ttm2-1* and *ttm1-1 ttm2-1* mutants exhibited similar but lower seed germination rates and reduced cotyledon greening in comparison to the wild type (Figure 6B–D), suggesting that TTM2 but not TTM1 has a role in ABA-regulated seed germination and early seedling development.

## 3. Discussion

Seed germination is one of the most important stages of plant growth and development, which is affected by multiple exogenous and endogenous factors [8,47,48]. Here, we focused on *TTM2*, a previously reported gene involved in plant biotic stress, whose function in plant growth and development is unclear [38]. Our data found that ABI4 represses *TTM2* expression during ABA-mediated seed germination. We first explored the expression of *TTM2* at various stages of seed germination based on public microarray data. The results displayed that the *TTM2* expression increased rapidly after the imbibition and germination stage (Figure 1A). We speculated that *TTM2* may be involved in the seed germination process. Since many events of germination are regulated by the incorporation of ABA and GA, two hormones that have antagonistic effects on the germination process. We examined the transcript level of *TTM2* under ABA and GA treatment, respectively. The experimental results revealed that ABA significantly represses the transcription of *TTM2*, but GA does not (Figure 1B and Figure S4). Further analysis showed that germination of the *ttm2* knockout mutant was hypersensitive to ABA, whereas *TTM2*-overexpressing plants were less insensitive to ABA (Figure 2). The results suggested that TTM2 is involved in ABA-mediated seed germination.

ABI4 acts as a key player in ABA-mediated germination [49,50]. We discovered that exogenous ABA significantly repressed *TTM2* expression in the wild type. However, the repression was significantly attenuated in the *abi4-1* mutant (Figure 3), suggesting that *TTM2* may act downstream of *ABI4*. ABI4, an active ABA signaling response transcription factor, has both transcriptional repression and activation effects [34]. It can bind to gene promoters CE1-liKE motif (CACCG) to regulate the transcription of some genes such as *ABI5*, *NCED6*, *Starch Branching Enzyme*, *Acyl-coenzyme A: Diacylglycerol Acyltransferase1*, and *CHOTTO1* responding to ABA signaling [33,35,51,52,53]. Further studies revealed that binding capacity still exists in degenerate bases of the CE1-liKE motif (CCAC) in the promoters of some genes [54], such as *CYCP707A1*, *CYCP707A2*, and *Vitamin C Defective* 2 [18,23]. We identified the CCAC box in the *TTM2* promoter (Figure 4A) and our results revealed that ABI4 binds to the *TTM2* promoter and inhibits the expression of *TTM2* (Figure 4B–E). Consistently, a recent study showed that ABI4 can bind thousands of gene promoters, including *TTM2* by using the ChIP-seq assay [55]. Furthermore, in germination experiments, the *ttm2-1* mutant rescued the ABA-insensitive phenotype of the *abi4-1* mutant (Figure 5), further supporting that ABI4 directly inhibited *TTM2* expression in response to ABA-dependent seed germination. In addition, we found that the transcription level of *TTM2* can be still repressed by ABA in either *abi3-4* or *abi5-1* (Figure 3 and Figure S5). Additionally, the *TTM2* promoter does not contain the core binding motifs of ABI3 and ABI5, which further validates that *TTM2* may not be regulated by ABI3/5 in ABA signaling. However, ABA-dependent transcription factors also contain many members, such as the MYB superfamily and WRKY superfamily [56,57,58]. And we found a large number of WRKY core motif W-boxes (TGAC) and MYB core sequences (TTGTTA) in the *TTM2* promoter. It means that *TTM2* may act as an essential target regulated by multiple aspects of ABA signaling, which deserves further consideration in the future.

The TTM family is an evolutionarily well-conserved protein family in eukaryotes. Until now, only a few reports have described the function of this family in plants [37,38,39,40,59]. Here, we found that *ttm1-1* mutants don^’^t show sensitivity to ABA during seed germination, and the sensitivity of the *ttm1-1 ttm2-1* double mutant to ABA was similar to that of the *ttm2-1* mutant in seed germination (Figure 6), which means there was non-redundant function in *TTM1* and *TTM2*. Interestingly, researchers have recently found that TTM1 is implicated in ABA-mediated leaf senescence [40]. Multiple mitogen-activated protein kinases (MPK1/3/4/6/7) phosphorylate the three main TTM1 sites (Ser10, Ser437, and Ser490), and the phosphorylation of Ser437 is essential for the role of TTM1 in leaf senescence [40]. TTM2 has extremely high sequence identity (65.7%) and similarity (92.3%) to TTM1 [37]. Amino acid sequence alignment showed that Ser437 and Ser490 are fully conserved in TTM1 and TTM2. Whether the regulation of MPK phosphorylation is also present in TTM2 protein in ABA-mediated seed germination also deserves further investigation.

In conclusion, our results indicate that TTM2 acts as a downstream factor of ABI4 in ABA-mediated seed germination and early seedling growth.

## 4. Materials and Methods

### 4.1. Plant Material and Growth Conditions

The mutant lines used in this study were obtained from the Arabidopsis Biological Resource Center. In this study, we used different *Arabidopsis thaliana* ecotypes as wild type, including Landsberg *erecta* (L*er*), Columbia (Col-0), or Wassilewskija (Ws). The mutants *ttm2-1* (SALK_145897) [38], *ttm2-2* (SALK_114669) [38], *abi4-1* (CS8104) [60], *ttm1-1* (SALK_079237) [37] were compared with Col-0. The mutant *abi3-4* (CS6130) [61] was compared with L*er*. The mutant *abi5-1* (CS8105) [19] was compared with Ws. The double mutant *abi4-1 ttm2-1* was obtained by hybridizing *abi4-1* and *ttm2-1* and was confirmed by PCR. The double mutant *ttm1-1 ttm2-1* was obtained by hybridizing *ttm1-1* and *ttm2-1* and was confirmed by PCR. The sterile Arabidopsis seeds were suspended in sterile water for three days at 4 °C after being surface disinfected with 5% bleach and cleaned with sterile water at least three times. Then the imbibed seeds were sown on 1/2 MS medium with 0.8% agarose and 1% sucrose and transferred to 23 °C for incubation under a 16 h light/8 h dark photoperiod with a 120 μmol m^–2^s^–1^ light setting. Primer sequences are listed in Table S1.

### 4.2. Production of Transgenic Plants

For generating the *ProTTM2::GUS* vectors, the *TTM2* promoter sequence covering the 2 kb upstream of the ATG start codon was amplified and cloned into the pCAMBIA1300-GUS vector. To construct *35S::TTM2-FLAG* vectors, the amplified CDS of *TTM2* was cloned into the pCAMBIA1300-FLAG vector controlled by the *35S* promoter. To construct *35S::ABI4-GFP* vectors, the amplified CDS of *ABI4* was cloned into the pCAMBIA1300-GFP vector controlled by the *35S* promoter. Then the vectors of *ProTTM2::GUS*, *35S::TTM2-FLAG*, and *35S::ABI4-GFP* were transformed into *Arabidopsis* Col-0,respectively, using *Agrobacterium*-mediated flower immersion [62]. The homozygous T3 plants were selected on a medium containing kanamycin and then verified by PCR and qRT-PCR. Primer sequences are listed in Table S1.

### 4.3. Determination of Germination Rate and Green Cotyledon

Germination rate and green cotyledons were determined following the methods of previous studies [63]. Briefly, the seeds were sterilized and imbibed at 4 °C for 3 days and then they were planted on 1/2 MS medium with or without ABA/GA_3_ (Solarbio Science Technology Co., Ltd., Beijing, China). Seed germination was followed for 8 days and the germination rate was measured by the appearance of radicles through the seed coat; green cotyledons were counted by the emergence of green cotyledons in seedlings at 8 d. Three biological duplicates of each experiment were run, and equivalent outcomes were achieved. For each treatment, more than 100 seeds were counted.

### 4.4. RNA Extraction and Quantitative Real-Time PCR

Total RNA was isolated from seeds or seedlings using TRIzol Reagent (TransGen Biotech, Beijing, China). The cDNA was synthesized by reverse transcription after the removal of DNA contamination (TransGen Biotech, Beijing, China). The qRT-PCR was performed in 96-well plates by adding SYBR Green I dye (Monad, Suzhou, China) and then placed on a Bio-Rad CFX96 device (Bio-Rad, Hercules, CA, USA) and programmed as follows: 95 °C/3 min for pre-denaturation, followed by 95 °C/15 s and 60 °C/30 s for 45 cycles. The *ACT2* gene was used as a reference gene. The experiments were duplicated at least three times biologically. The primer sequences are listed in Table S1.

### 4.5. Electrophoresis Mobility Shift Assay (EMSA)

The CDS of *ABI4* was integrated into the pET28a and transformed into *E. coli* BL21 strain in order to produce the ABI4 protein. The biotin-labeled oligonucleotide probe and purified ABI4 protein were utilized for EMSA determination. According to the manufacturer’s protocol, experiments were conducted using the Light Shift chemiluminescent EMSA kit (Thermo Scientific, Waltham, MA, USA). The probes used in the experiments are listed in Table S1.

### 4.6. Chromatin Immunoprecipitation (ChIP)-qPCR Assay

Details of ChIP assay are reported in previous studies [64]. The *35S::ABI4-GFP* transgenic plants germinating for 3 days were fixed with formaldehyde, then the tissues were crushed, and the chromatin was separated and ultra-sonicated. Anti-GFP antibody was used for immunoprecipitation. ChIP-qPCR was performed on DNA fragments enriched with Anti-GFP, while non-immunoprecipitated sonicated chromatin was utilized as input control. The experiment used *UBQ5* as an internal control. The results of the experiment were similar in three independent biological replicates. The primers are listed in Table S1.

### 4.7. Dual Luciferase Assays

In a dual luciferase (dual-LUC) assay, the *ABI4* CDS was inserted into the pCAMBIA1300-GFP vector under the control of a *35S* promoter to generate an effector construct and transformed the recombinant construct into *Agrobacterium tumefaciens* strain GV3101. To generate the reporter construct, the *TTM2* promoter region was cloned separately into the pGreenII 0800-LUC and the recombinant vectors were transformed into *Agrobacterium rhizogenes* strain C58C1. Pseudomonas hybrids were infected with *Nicotiana benthamiana* leaves. Following the manufacturer’s instructions, the fluorescence values were read on a LUC fluorescence detector (Promega, Madison, WI, USA) by using a dual fluorogenic enzyme reporter gene assay kit (Beyotime Biotechnology, Shanghai, China). The primers are listed in Table S1.

### 4.8. GUS Staining

GUS assays were carried out using a staining kit (Coolaber, Beijing, China). The staining buffer was used to incubate *ProTTM2::GUS* transgenic plant tissues at 37 °C for 8–12 h. After termination of the reaction, the tissue was cleared with 70% ethanol and imaged.

## 5. Conclusions

In summary, our work uncovers a novel function of *TTM2*, which is involved in ABA-mediated seed germination and early seedling development and is transcriptionally repressed by ABI4. In contrast, TTM1, a homologous protein of TTM2, is found to have no apparent function in this process, further suggesting that they are involved in independent biological processes. Our study provides new insights into further understanding of seed germination regulatory mechanisms.

Accession Numbers: For the genes described in this article, the *Arabidopsis* Genome Initiative numbers are as follows: TTM2 (At1G26190), TTM1 (AT1G73980), ABI4 (AT2G40220), ACTIN2 (AT3G18780), ABI3 (AT3G24650), ABI5 (AT2G36270), and UBQ5 (AT3G62250).

## Data Availability

The data are present within the article or the Supplementary Materials.

## Supplementary Materials

The supporting information can be downloaded at: https://www.mdpi.com/article/10.3390/ijms24108994/s1.

## References

[1] Finch-Savage W.E., Footitt S. (2017). Seed dormancy cycling and the regulation of dormancy mechanisms to time germination in variable field environments. J. Exp. Bot..

[2] Neé G., Xiang Y., Soppe W.J. (2017). The release of dormancy, a wake-up call for seeds to germinate. Curr. Opin. Plant Biol..

[3] Shu K., Liu X.D., Xie Q., He Z.H. (2016). Two Faces of One Seed: Hormonal Regulation of Dormancy and Germination. Mol. Plant..

[4] Shinomura T., Nagatani A., Chory J., Furuya M. (1994). The lnduction of Seed Germination in Arabidopsis tbaliana 1s Regulated Principally by Phytochrome B and Secondarily by Phytochrome A. Plant Physiol..

[5] Lechowska K., Kubala S., Wojtyla A., Nowaczyk G., Quinet M., Lutts S., Garnczarska M. (2019). New Insight on Water Status in Germinating *Brassica napus* Seeds in Relation to Priming-Improved Germination. Int. J. Mol. Sci..

[6] Teixeira S.B., Pires S.N., Ávila G.E., Silva B.E.P., Schmitz V.N., Deuner C., Da Silva Armesto R., Da Silva Moura D., Deuner S. (2021). Application of vigor indexes to evaluate the cold tolerance in rice seeds germination conditioned in plant extract. Sci. Rep..

[7] Giraudat J.L.A.J. (1998). Abscisic Acid Signal Transduction. Annu. Rev. Plant Physiol..

[8] Yang L., Jiang Z., Liu S., Lin R. (2020). Interplay between REVEILLE1 and RGA-LIKE2 regulates seed dormancy and germination in Arabidopsis. New Phytol..

[9] Grappin P., Bouinot D., Sotta B., Miginiac E., Jullien M. (2000). Control of seed dormancy in Nicotiana plumbaginifolia: Post-imbibition abscisic acid synthesis imposes dormancy maintenance. Planta.

[10] Zhao H., Nie K., Zhou H., Yan X., Zhan Q., Zheng Y., Song C.P. (2020). ABI5 modulates seed germination via feedback regulation of the expression of the PYR/PYL/RCAR ABA receptor genes. New Phytol..

[11] Li Z., Sheerin D.J., von Roepenack-Lahaye E., Stahl M., Hiltbrunner A. (2022). The phytochrome interacting proteins ERF55 and ERF58 repress light-induced seed germination in Arabidopsis thaliana. Nat. Commun..

[12] Okamoto M., Kuwahara A., Seo M., Kushiro T., Asami T., Hirai N., Kamiya Y., Koshiba T., Nambara E. (2006). CYP707A1 and CYP707A2, Which Encode Abscisic Acid 8′-Hydroxylases, Are Indispensable for Proper Control of Seed Dormancy and Germination in Arabidopsis. Plant Physiol..

[13] Lv Y., Pan J., Wang H., Reiter R.J., Li X., Mou Z., Zhang J., Yao Z., Zhao D., Yu D. (2021). Melatonin inhibits seed germination by crosstalk with abscisic acid, gibberellin, and auxin in Arabidopsis. J. Pineal Res..

[14] Manfre A.J., Lanni L.M., Marcotte W.R. (2006). The Arabidopsis Group 1 Late Embryogenesis Abundant Protein ATEM6 Is Required for Normal Seed Development. Plant Physiol..

[15] He Z., Wu J., Sun X., Dai M. (2019). The Maize Clade A PP2C Phosphatases Play Critical Roles in Multiple Abiotic Stress Responses. Int. J. Mol. Sci..

[16] Hauser F., Waadt R., Schroeder J.I. (2011). Evolution of Abscisic Acid Synthesis and Signaling Mechanisms. Curr. Biol..

[17] Koornneef M., Reuling G., Karssen C.M. (1984). The isolation and characterization of abscisic acid-insensitive. Physiol. Plant..

[18] Shu K., Zhang H., Wang S., Chen M., Wu Y., Tang S., Liu C., Feng Y., Cao X., Xie Q. (2013). ABI4 regulates primary seed dormancy by regulating the biogenesis of abscisic acid and gibberellins in arabidopsis. PLoS Genet..

[19] Finkelstein R.R., Lynch T.J. (2000). The Arabidopsis abscisic acid response gene ABI5 encodes a basic leucine zipper transcription factor. Plant Cell.

[20] Finkelstein R.R., Somerville C.R. (1990). Three Classes of Abscisic Acid (ABA)-lnsensitive Mutations of Arabidopsis Define Genes that Control Overlapping Subsets of ABA Responses. Plant Physiol..

[21] Acevedo-Hernández G.J., León P., Herrera-Estrella L.R. (2005). Sugar and ABA responsiveness of a minimal RBCS light-responsive unit is mediated by direct binding of ABI4. Plant J..

[22] Xie Y., Mao Y., Duan X., Zhou H., Lai D., Zhang Y., Shen W. (2016). Arabidopsis HY1-Modulated Stomatal Movement: An Integrative Hub Is Functionally Associated with ABI4 in Dehydration-Induced ABA Responsiveness. Plant Physiol..

[23] Kakan X., Yu Y., Li S., Li X., Huang R., Wang J. (2021). Ascorbic acid modulation by ABI4 transcriptional repression of VTC2 in the salt tolerance of Arabidopsis. BMC Plant Biol..

[24] Shu K., Chen Q., Wu Y., Liu R., Zhang H., Wang S., Tang S., Yang W., Xie Q. (2015). ABSCISIC ACID-INSENSITIVE 4 negatively regulates flowering through directly promoting Arabidopsis Flowering Locus C transcription. J. Exp. Bot..

[25] Feng C.Z., Chen Y., Wang C., Kong Y.H., Wu W.H., Chen Y.F. (2014). Arabidopsis RAV1 transcription factor, phosphorylated by SnRK2 kinases, regulates the expression ofABI3,ABI 4, and ABI5 during seed germination and early seedling development. Plant J..

[26] Chandrasekaran U., Luo X., Zhou W., Shu K. (2020). Multifaceted Signaling Networks Mediated by Abscisic Acid Insensitive 4. Plant. Commun..

[27] Xiao S., Jiang L., Wang C., Ow D.W. (2021). Arabidopsis OXS3 family proteins repress ABA signaling through interactions with AFP1 in the regulation ofABI4 expression. J. Exp. Bot..

[28] Zhu Y., Hu X., Duan Y., Li S., Wang Y., Rehman A.U., He J., Zhang J., Hua D., Yang L. (2020). The Arabidopsis Nodulin Homeobox Factor AtNDX Interacts with AtRING1A/B and Negatively Regulates Abscisic Acid Signaling. Plant Cell.

[29] Zhao Y., Ai X., Wang M., Xiao L., Xia G. (2016). A putative pyruvate transporter TaBASS2 positively regulates salinity tolerance in wheat via modulation of ABI4 expression. BMC Plant Biol..

[30] Huang Y., Feng C., Ye Q., Wu W., Chen Y. (2016). Arabidopsis WRKY6 Transcription Factor Acts as a Positive Regulator of Abscisic Acid Signaling during Seed Germination and Early Seedling Development. PLoS Genet..

[31] Lee K., Lee H.G., Yoon S., Kim H.U., Seo P.J. (2015). The Arabidopsis MYB96 Transcription Factor Is a Positive Regulator ofABSCISIC ACID-INSENSITIVE4 in the Control of Seed Germination. Plant Physiol..

[32] Greco M., Chiappetta A., Bruno L., Bitonti M.B. (2012). In Posidonia oceanica cadmium induces changes in DNA methylation and chromatin patterning. J. Exp. Bot..

[33] Bossi F., Cordoba E., Dupré P., Mendoza M.S., Román C.S., León P. (2009). The Arabidopsis ABA-INSENSITIVE (ABI) 4 factor acts as a central transcription activator of the expression of its own gene, and for the induction of ABI5 and SBE2.2 genes during sugar signaling. Plant J..

[34] Wind J.J., Peviani A., Snel B., Hanson J., Smeekens S.C. (2013). ABI4: Versatile activator and repressor. Trends Plant Sci..

[35] Yamagishi K., Tatematsu K., Yano R., Preston J., Kitamura S., Takahashi H., McCourt P., Kamiya Y., Nambara E. (2009). CHOTTO1, a Double AP2 Domain Protein of Arabidopsis thaliana, Regulates Germination and Seedling Growth Under Excess Supply of Glucose and Nitrate. Plant Cell Physiol..

[36] Iyer L.M., Aravind L. (2002). The catalytic domains of thiamine triphosphatase and CyaB-like adenylyl cyclase define a novel superfamily of domains that bind organic phosphates. BMC Genom..

[37] Ung H., Karia P., Ebine K., Ueda T., Yoshioka K., Moeder W. (2017). Triphosphate Tunnel Metalloenzyme Function in Senescence Highlights a Biological Diversification of This Protein Superfamily. Plant Physiol..

[38] Ung H., Moeder W., Yoshioka K. (2014). Arabidopsis Triphosphate Tunnel Metalloenzyme2 Is a Negative Regulator of the Salicylic Acid-Mediated Feedback Amplification Loop for Defense Responses. Plant Physiol..

[39] Moeder W., Garcia-Petit C., Ung H., Fucile G., Samuel M.A., Christendat D., Yoshioka K. (2013). Crystal structure and biochemical analyses reveal that the Arabidopsis triphosphate tunnel metalloenzyme AtTTM3 is a tripolyphosphatase involved in root development. Plant J..

[40] Karia P., Yoshioka K., Moeder W. (2021). Multiple phosphorylation events of the mitochondrial membrane protein TTM1 regulate cell death during senescence. Plant J..

[41] Klepikova A.V., Kasianov A.S., Gerasimov E.S., Logacheva M.D., Penin A.A. (2016). A high resolution map of the Arabidopsis thaliana developmental transcriptome based on RNA-seq profiling. Plant J..

[42] Liu H., Guo S., Lu M., Zhang Y., Li J., Wang W., Wang P., Zhang J., Hu Z., Li L. (2019). Biosynthesis of DHGA12 and its roles in Arabidopsis seedling establishment. Nat. Commun..

[43] Gubler F., AMillar A., VJacobsen J. (2005). Dormancy release, ABA and pre-harvest sprouting Frank Gubler1,2, Anthony A Millar2 and John V Jacobsen1. Curr. Opin. Plant Biol..

[44] Zhao H., Zhang Y., Zheng Y. (2022). Integration of ABA, GA, and light signaling in seed germination through the regulation of ABI5. Front. Plant Sci..

[45] Skubacz A., Daszkowska-Golec A., Szarejko I. (2016). The Role and Regulation of ABI5 (ABA-Insensitive 5) in Plant Development, Abiotic Stress Responses and Phytohormone Crosstalk. Front. Plant Sci..

[46] Söderman E.M.E.M., Brocard I.M.I.M., Lynch T.J.T.J., Finkelstein R.R.R.R. (2000). Regulation and Function of the Arabidopsis ABA-insensitive4 Gene in Seed and Abscisic Acid Response Signaling Networks1. Plant Physiol..

[47] Hossain A., Teixeira Da Silva J.A., Lozovskaya M.V., Zvolinsky V.P. (2012). High temperature combined with drought affect rainfed spring wheat and barley in South-Eastern Russia: I. Phenology and growth. Saudi J. Biol. Sci..

[48] Boter M., Calleja-Cabrera J., Carrera-Castaño G., Wagner G., Hatzig S.V., Snowdon R.J., Legoahec L., Bianchetti G., Bouchereau A., Nesi N. (2019). An Integrative Approach to Analyze Seed Germination in Brassica napus. Front. Plant Sci..

[49] Xu Z., Kim D.H., Hwang I. (2013). ABA homeostasis and signaling involving multiple subcellular compartments and multiple receptors. Plant Cell Rep..

[50] Cutler S.R., Rodriguez P.L., Finkelstein R.R., Abrams S.R. (2010). Abscisic acid: Emergence of a core signaling network. Annu. Rev. Plant Biol..

[51] Shu K., Chen Q., Wu Y., Liu R., Zhang H., Wang P., Li Y., Wang S., Tang S., Liu C. (2016). ABI4 mediates antagonistic effects of abscisic acid and gibberellins at transcript and protein levels. Plant J..

[52] Yang Y., Yu X., Song L., An C. (2011). ABI4 Activates DGAT1 Expression in Arabidopsis Seedlings during Nitrogen Deficiency. Plant Physiol..

[53] Nambara E., Suzuki M., Abrams S., McCarty D.R., Kamiya Y., McCourt P. (2002). A Screen for Genes That Function in Abscisic Acid Signaling in Arabidopsis thaliana. Genetics.

[54] Koussevitzky S., Nott A., Mockler T.C., Hong F., Sachetto-Martins G., Surpin M., Lim J., Mittler R., Chory J. (2007). Signals from chloroplasts converge to regulate nuclear gene expression. Science.

[55] Luo X., Xu J., Zheng C., Yang Y., Wang L., Zhang R., Ren X., Wei S., Aziz U., Du J. (2022). Abscisic acid inhibits primary root growth by impairing ABI4-mediated cell cycle and auxin biosynthesis. Plant Physiol..

[56] Jiang W., Yu D. (2009). Arabidopsis WRKY2 transcription factor mediates seed germination and postgermination arrest of development by abscisic acid. BMC Plant Biol..

[57] Yang B., Song Z., Li C., Jiang J., Zhou Y., Wang R., Wang Q., Ni C., Liang Q., Chen H. (2018). RSM1, an Arabidopsis MYB protein, interacts with HY5/HYH to modulate seed germination and seedling development in response to abscisic acid and salinity. PLoS Genet..

[58] Rushton D.L., Tripathi P., Rabara R.C., Lin J., Ringler P., Boken A.K., Langum T.J., Smidt L., Boomsma D.D., Emme N.J. (2012). WRKY transcription factors: Key components in abscisic acid signalling. Plant Biotechnol. J..

[59] Lorenzo-Orts L., Witthoeft J., Deforges J., Martinez J., Loubéry S., Placzek A., Poirier Y., Hothorn L.A., Jaillais Y., Hothorn M. (2019). Concerted expression of a cell cycle regulator and a metabolic enzyme from a bicistronic transcript in plants. Nat. Plants.

[60] Finkelstein R.R., Wang M.L., Lynch T.J., Rao S., Goodman H.M. (1998). The Arabidopsis abscisic acid response locus ABI4 encodes an APETALA 2 domain protein. Plant Cell..

[61] Giraudat J., Hauge B.M., Valon C., Smalle J., Parcy F., Goodman H.M. (1992). lsolation of the Arabidopsis AB13 Gene by Positional Cloning. Plant Cell.

[62] Hong L., Yan D., Liu W., Chen H., Lu Y. (2014). TIME FOR COFFEE controls root meristem size by changes in auxin accumulation in Arabidopsis. J. Exp. Bot..

[63] Huang Y., Sun M., Ye Q., Wu X., Wu W., Chen Y. (2017). Abscisic Acid Modulates Seed Germination via ABA INSENSITIVE5-Mediated PHOSPHATE1. Plant Physiol..

[64] Yuan T., Xu H., Zhang Q., Zhang L., Lu Y. (2018). The COP1 Target SHI-RELATED SEQUENCE5 Directly Activates Photomorphogenesis-Promoting Genes. Plant Cell.

