# Supplementary material for: Triphosphate Tunnel Metalloenzyme 2 Acts as a Downstream Factor of ABI4 in ABA-Mediated Seed Germination
Source: Int J Mol Sci. 2023 May 19;24(10):8994. doi: 10.3390/ijms24108994 (PMC10218978; doi:10.3390/ijms24108994)
Supplement: Supplementary file 1 [file ijms-24-08994-s001.zip › ijms-2321105-supplementary.pdf]

Supplementary Materials

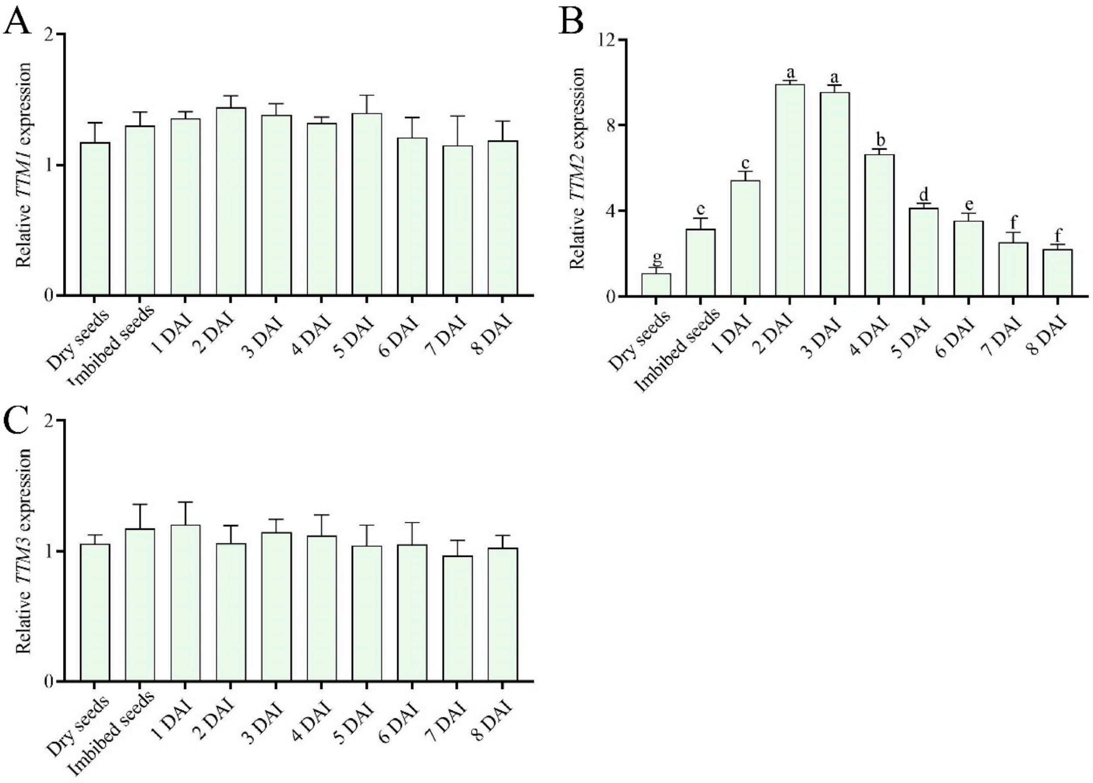

Figure S1. Analysis of *TTM1*, *TTM2*, and *TTM3* transcription levels by qRT-PCR. **(A-C)** The transcription levels of *TTM1*(A), *TTM2* (B), and *TTM3* (C) during seed germination. The wild-type (WT) seeds were soaked at 4°C for 3 days in water and grown on 1/2 Murashige & Skoog (MS) medium for 1-8 days. DAI, days after imbibition. The *ACT2* gene was applied as an internal control. Expression levels were normalized to dry seeds, which was set at 1. Data are shown as mean ± SD (n = 3). Various letters represent significant differences at $P < 0.05$ by one-way ANOVA and Tukey's multiple comparison test.

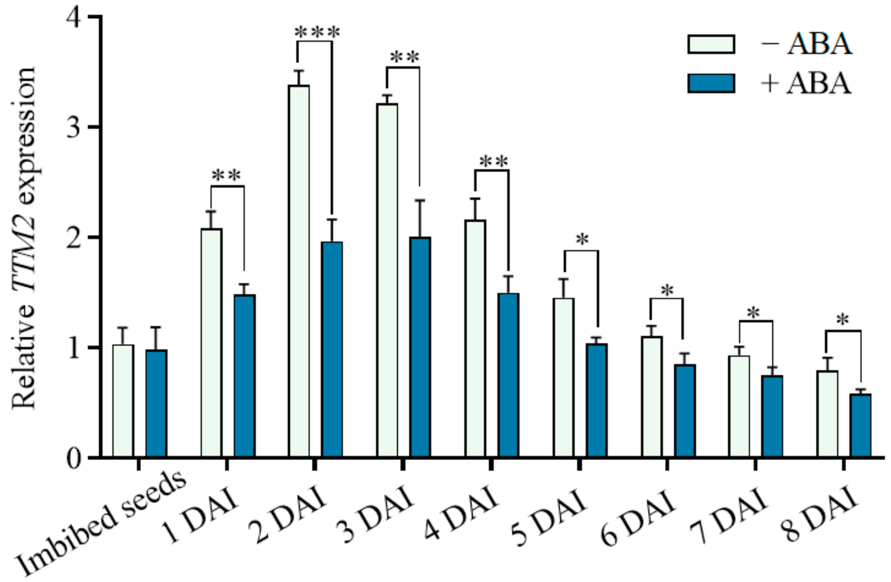

Figure S2. The qRT-PCR to determine the expression of *TTM2*. Imbibed seeds of the wild type were planted in 1/2 MS medium (−ABA) or 1/2 MS medium containing 0.5 μM ABA (+ABA) for 1-8 days, followed by RNA extraction. The *ACT2* gene was applied as an internal control. Expression levels were normalized to those of untreated (−ABA) imbibed seeds, which was set at 1. Data are shown as mean ± SD (n = 3). Asterisk denotes a statistically significant difference compared with their -ABA at indicated times. *, $P < 0.05$; **, $P < 0.01$; ***, $P < 0.001$ (Student's *t*-test).

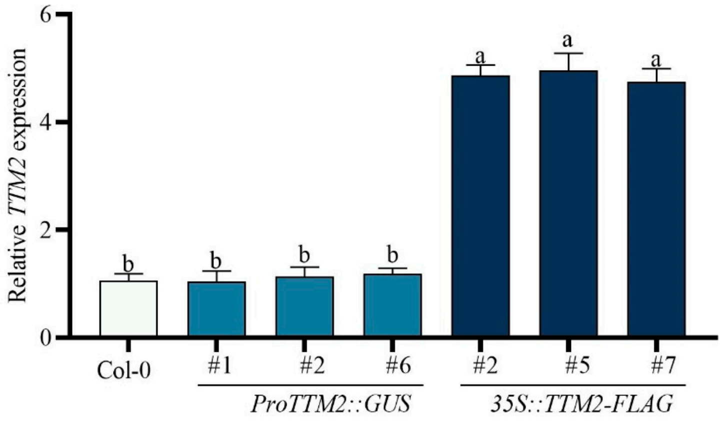

Figure S3. Validation of *ProTTM2::GUS* and *35S::TTM2-FLAG* transgenic lines. The qRT-PCR to determine the expression of *TTM2*. The *ACT2* gene was applied as an internal control. Expression levels were normalized to Col-0 plants, which was set at 1. Data are means ± SD (n = 3). Various letters represent significant differences at P < 0.05 by one-way ANOVA with Tukey's multiple comparison test.

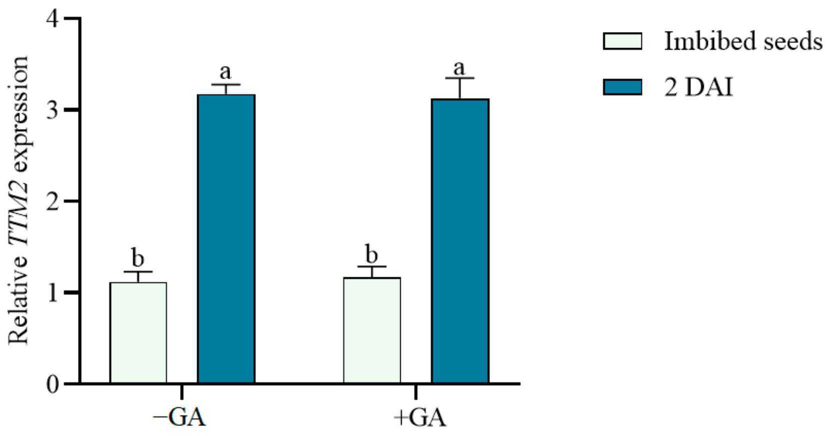

Figure S4. The exogenous GA treatment did not affect the *TTM2* expression. The qRT-PCR to determine the expression of *TTM2*. Imbibed wild-type seeds were planted on 1/2 MS medium (−GA) or 1/2 MS medium containing 10μM GA_3 (+GA) for 2 days, followed by RNA extraction. The *ACT2* gene was applied as an internal control. Expression levels were normalized to those of untreated (−GA) imbibed seeds, which was set at 1. Data are shown as mean ± SD ($n = 3$). Various letters represent significant differences at $P < 0.05$ by one-way ANOVA with Tukey's multiple comparison test.

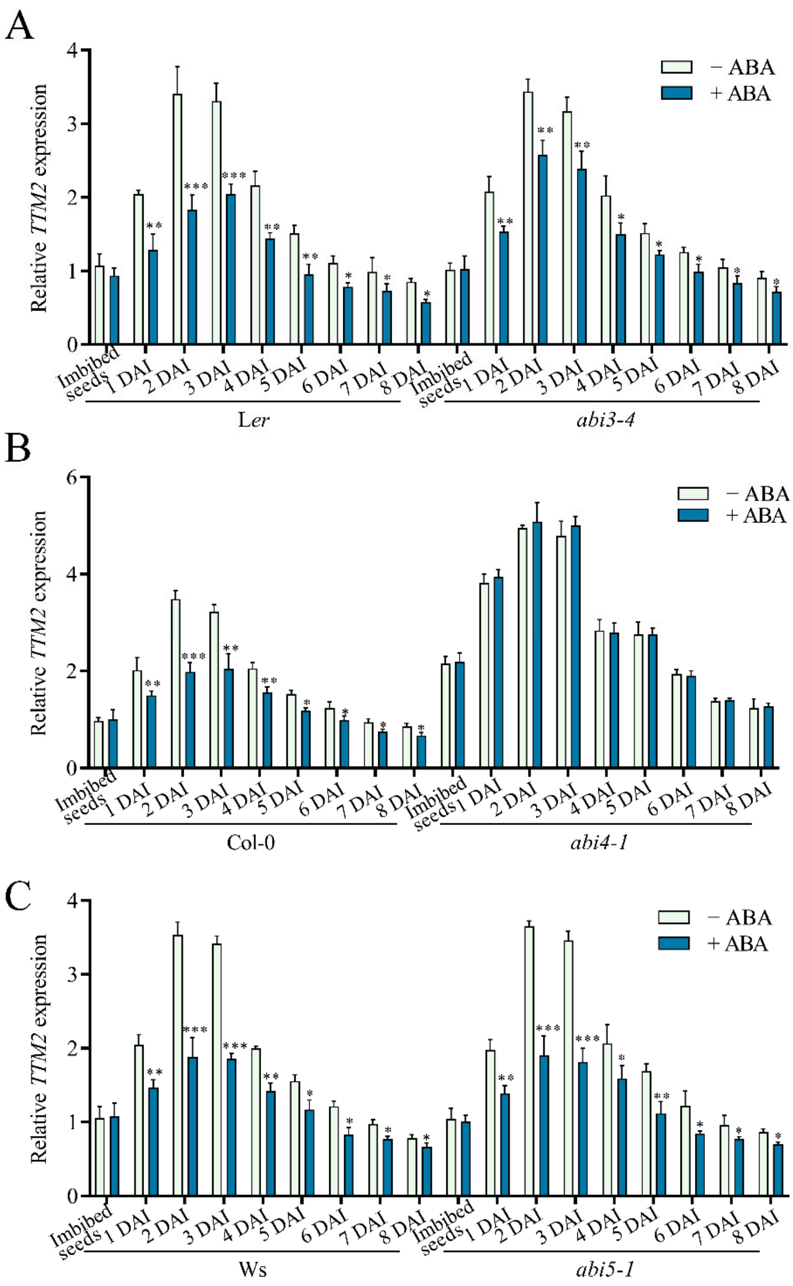

Figure S5. *TTM2* expression in response to ABA in *abi3-4*, *abi4-1*, and *abi5-1*. **(A)** *TTM2* expression in Landsberg erecta (L*er*) and *abi3-4*. **(B)** *TTM2* expression in Columbia (Col-0) and *abi4-1*. **(C)** *TTM2* expression in Wassilewskija (Ws) and *abi5-1*. Imbibed seeds of different genotypes were germinated on 1/2 MS medium (−ABA) or 1/2 MS medium with 0.5 µM ABA (+ABA) for 1-8 days. Harvested plants were subjected to RNA extraction and qRT-PCR analysis. The *ACT2* gene was applied as an internal control. Expression levels were normalized to those of untreated (−ABA) imbibed seeds, which was set at 1. Data are shown as mean ± SD (n = 3). Asterisk denotes a statistically significant difference compared with their -ABA at indicated times. *, $P < 0.05$; **, $P < 0.01$; ***, $P < 0.001$ (Student's *t*-test).

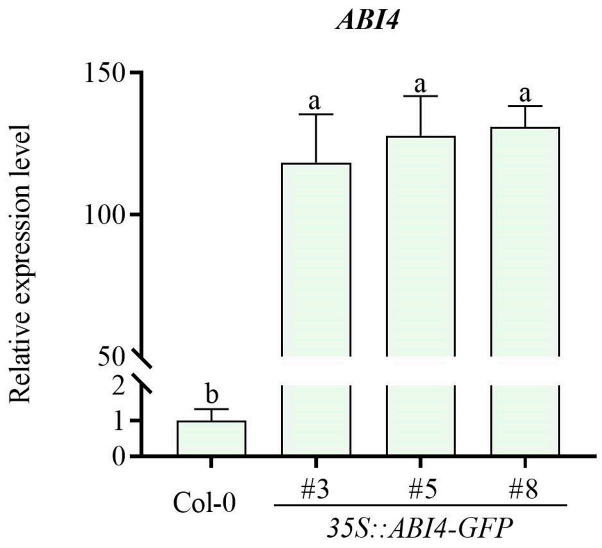

Figure S6. Validation of *35S::ABI4-GFP* transgenic lines. The qRT-PCR to determine the *ABI4* expression. The *ACT2* gene was applied as a control. Expression levels were normalized to Col-0 plants, which was set at 1. Data are means ± SD (*n*=3). Various letters represent significant differences at $P < 0.05$ by one-way ANOVA with Tukey's multiple comparison test.

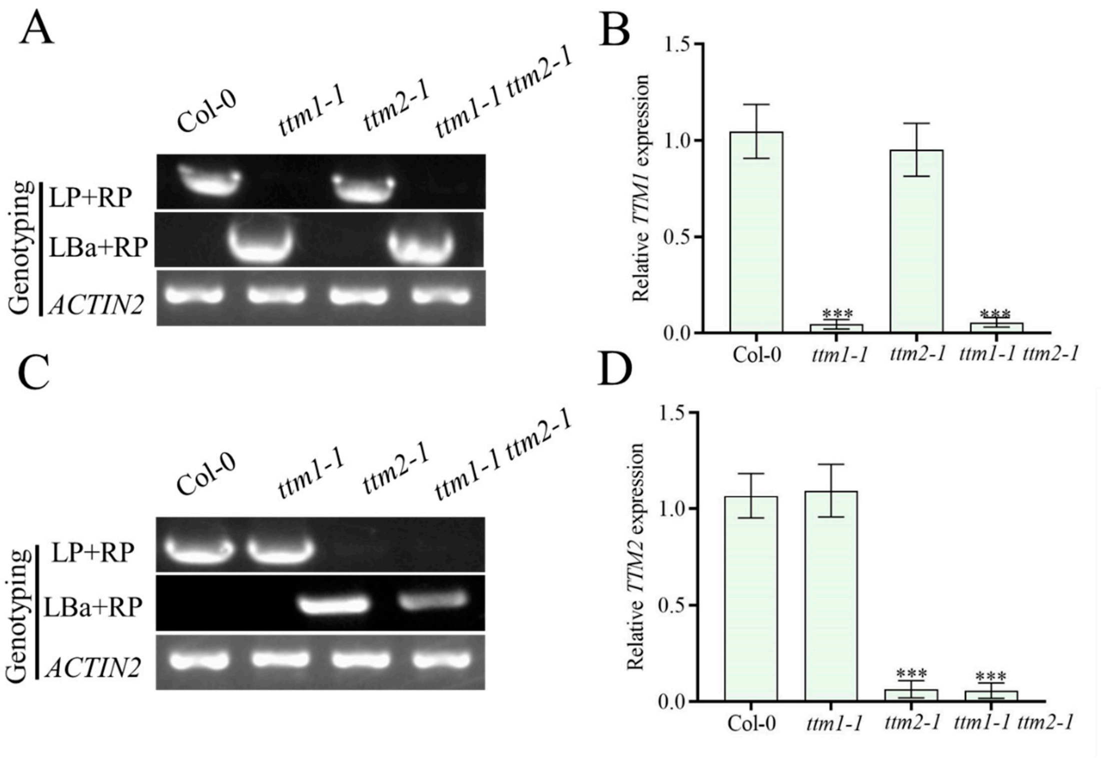

Figure S7. The verification of plant genotype in Col-0, *ttm1-1*, *ttm2-1*, and *ttm1-1 ttm2-1*. (A) The T-DNA insertion of *ttm1-1* was determined by genotyping in Col-0, *ttm1-1*, *ttm2-1*, and *ttm1-1 ttm2-1.* (B) The determination of *TTM1* expression by qRT-PCR in Col-0, *ttm1-1*, *ttm2-1*, and *ttm1-1 ttm2-1*. (C) The T-DNA insertion of *ttm2-1* was determined by genotyping in Col-0, *ttm1-1*, *ttm2-1*, and *ttm1-1 ttm2-1.* (D) The determination of *TTM2* expression by qRT-PCR in Col-0, *ttm1-1*, *ttm2-1*, and *ttm1-1 ttm2-1.* Expression levels were normalized to Col-0, which was set at 1. Data are shown as mean ± SD (n = 3). Asterisk denotes a statistically significant difference compared with Col-0. ***, $P < 0.001$ (Student's *t*-test).

Table S1. List of the primers used in this study

Primer name	Sequence (5' to 3')
**Genomic DNA PCR**	
LBa1	ATTTTGCCGATTTCGGAAC
ttm1-1-LP	TTCTGCTCATGCTTTGATTTG
ttm1-1-RP	AGCAAGGGTGATTAATCTGGG
ttm2-1-LP	TATGTCCATCAGAAAGGACCG
ttm2-1-RP	CTTCTGGTGCTGGAAAGACTG
ttm2-2-LP	GTAATGCGTGACGTGATTGTG
ttm2-2-RP	GTATCCGTAACCCATTCCTGG
abi4-1-F	GCCACCGTAGGAGGAGGATC
abi4-1-R	TGTTGGAATTGTCCCATCTGGA
ACT2F	TCGATCTAAGTTGACCGATC
ACT2R	ACCTCTCTTGGATTGTGCTT
**Molecular Cloning**	
35S	CCACGTCTTCAAAGCAAGTGGATTGATGTG
35S-TTM2-F	CAAATCTATCTCTCTCGAGATGGGTCAAGACAGCAATGG
35S-TTM2-R	TCGTGGTCCTTATAATCTTGCCGCTTGTTAATGTAGC
1300F	TGTGTGGAATTGTGAGCGGATAAC
TTM2-GUS-F	TCGGTACCCGGGGATCCTATAAATATAAATTTCTAGT
TTM2-GUS-R	CCCTCAGATCTACCATTATGAACAATAATCTGCAAA
35S-ABI4-F	CCGGGCTCGAGAAGCTTATGGACCCTTTAGCTTCCC
35S-ABI4-R	GTCGACTCTAGAGGATCATAGAATTCCCCCAAGATGG
T7	TAATACGACTCACTATAGG
pET28a-ABI4-F	AGCAAATGGGTCGCGGAATGGACCCTTTAGCTTCCC
pET28a-ABI4-R	CGGAGCTCGAATTCGGAATAGAATTCCCCCAAGATGG
TTM2-LUC-F	GGTACCGGGCCCCCCCTCGAGTATAAATATAAATTTCTAGT
TTM2-LUC-R	TCGATACCGTCGACCTCGAGTATGAACAATAATCTGCAAA
**RT-qPCR**	
ACT2-F	TAACAGGGAGAAGATGACTCAGATCA
ACT2-R	AAGATCAAGACGAAGGATAGCATGAG
TTM2-F	GAAATCACGACTCGCCAGCG
TTM2-R	GCTCTGCTGTGTTGGAGAGT
ABI4-F	CTCGCAATGTCAAGATCCATTGGC
ABI4-R	TTACTCGCCGCACTGAAGTCAC
TTM1-F	TTCCAAGATCCCGAACAGGA
TTM1-R	ACCAGTACGACCTCTTCCAT

TTM3-F	AGTCACCGGCTAAGCTCTCA
TTM3-R	ACACGCTCTGGTTCCTCTGT
**ChIP-qPCR**	
TTM2-P1F	GTTTTATTATTACCTATGAC
TTM2-P1R	TTGATGACAGTTTTTGGTTTTTT
TTM2-P2F	AAAACTGTCATCAATAAAAA
TTM2-P2R	CTCTCTTCACTTCACGACCC
TTM2-P3F	GAAGTCAGAGAAAACCAAAA
TTM2-P3R	CATAGGCTTATGACTTATGA
TTM2-P4F	TAAATTGGTTCTCATTCATT
TTM2-P4R	GGGAGAGCGATTTGTAGAG
UBQ5-F	AGAAGATCAAGCACAAGCAT
UBQ5-R	CAGATCAAGCTTCAACTCCT
**EMSA Probe**	
TTM2-F	AAAATTGCACTCAAGAACATGTGGATCCTCGTCACATTCA C TCATAAGTCATAA
TTM2-R	TTATGACTTATGAGTGAATGTGACGAGGATCCACATGTTCT TGAGTGCAATTTT
